# Letter to the Editor: Unlocking Better Keloid Treatment: Corticosteroid, 5‐Fluorouracil, and Hyaluronidase vs. Corticosteroid Alone—A Randomized Comparative Study

**DOI:** 10.1002/hsr2.71356

**Published:** 2025-10-06

**Authors:** Asifa Zardari

**Affiliations:** ^1^ Liaquat University of Medical and Health Sciences Jamshoro Sindh Pakistan

Dear Editor,

I read the article titled “Unlocking Better Keloid Treatment: Corticosteroid, 5‐Fluorouracil, and Hyaluronidase versus Corticosteroid Alone—A Randomized Comparative Study” by Ehsani et al. with great interest. The study by Ehsani et al. introduces an innovative approach for treating keloid scars through a triple‐drug intralesional injection combining triamcinolone acetonide, 5‐fluorouracil (5‐FU), and hyaluronidase [1]. Their findings highlight superior outcomes in lesion height reduction, pliability, and Modified Vancouver Scar Scale (MVSS) scores compared to corticosteroid monotherapy. The biological rationale for adding hyaluronidase due to its potential to mitigate fibroblast proliferation and enhance drug spread adds depth to the study's clinical relevance. However, the inability to isolate hyaluronidase's independent effect due to the absence of a steroid + 5‐FU comparison group needs further discussion.

Methodologically, the trial presents several considerations, primarily due to its small sample size of sixteen participants (*n* = 16), which affects statistical power and generalizability. Although nonparametric tests were appropriately applied, the lack of reported effect sizes weakens the interpretability of the results [2]. Furthermore, the single‐blinded design, with patients aware of their treatment group, introduces performance bias that may affect subjective measures such as pain and pruritus [3]. While there were positive trends in these outcomes, they did not reach statistical significance, likely due to both small sample size and potential placebo effects.

Biological confounders also emerge in this trial. Including hyaluronidase seems logical due to its role in modulating interleukin‐1 and remodeling tissue; however, its effect is hard to separate from the well‐established effects of 5‐FU and triamcinolone [4]. Furthermore, the authors detected better outcomes in color change, but differences in initial scar pigmentation between groups undermine this finding. Moreover, the short follow‐up duration of 2 months is insufficient to assess the risk of keloid recurrence, which is a key consideration in evaluating any scar therapy [5].

To strengthen future research, larger multicenter trials with extended follow‐up are needed. A multi‐arm design isolating the effects of individual agents would provide greater clarity, while a double‐blinded methodology could minimize bias and improve validity. Stratification by lesion site and prior treatment history may also yield more refined insights. Despite its limitations, this study offers an encouraging step toward reducing the burden of keloids and underscores the promise of hyaluronidase as a potentially transformative therapeutic agent.

## Author Contributions

**Asifa Zardari:** writing – original draft, conceptualization, investigation, visualization, writing – review and editing.

## Conflicts of Interest

The author declares no conflicts of interest.

## Data Availability

The author has nothing to report.
